# Influence of Selected Factors on the Survival Assessment and Detection of *Giardia intestinalis* DNA in Axenic Culture

**DOI:** 10.3390/pathogens12020316

**Published:** 2023-02-14

**Authors:** Małgorzata Smoguła, Roland Wesołowski, Marta Pawłowska, Celestyna Mila-Kierzenkowska

**Affiliations:** Department of Medical Biology and Biochemistry, Ludwik Rydygier Collegium Medicum in Bydgoszcz, Nicolaus Copernicus University in Toruń, 87-100 Toruń, Poland

**Keywords:** *Giardia intestinalis*, microscopic method, real-time PCR method

## Abstract

*Giardia intestinalis* is one of the most common food-borne protozoa. The sensitivity of pathogens to physical and chemical factors is the basis for developing measures to reduce the incidence of the population. Several methods are available to detect the presence of *G. intestinalis*. The study determines the influence of 22 selected factors on the survival assessment and detection of *G. intestinalis* DNA in trophozoites axenically cultured. The influence of a given factor on the test result was observed in the case of 17 factors (77.3%) in the microscopic method, while only in the case of 3 (13.6%) substances in the real-time PCR method. Prevention of *G. intestinalis* infections, e.g., by ensuring food and water safety, is a crucial issue affecting public health. The experiment was conducted on trophozoites as the first approach. It is necessary to continue research and observe the epidemiological situation. In future studies, the impact of the studied factors on the survival assessment and detection of *Giardia intestinalis* DNA in axenically cultured cysts should be determined.

## 1. Introduction

*Giardia intestinalis* (syn. *Giardia lamblia* and *Giardia duodenalis*) is a protozoan parasite found in the small intestine of humans and some animals. The prevalence of *Giardia* infection ranges from about 2–5% in the industrialized world to 20–30% in low- and middle-income countries, with children tending to be infected more frequently than adults [1]. Infection with this flagellate usually occurs after consuming food or water containing *Giardia* cysts or directly via the fecal–oral route [2]. *Giardiasis* may be asymptomatic or symptomatic, acute or chronic. The clinical picture of giardiasis is greatly influenced by the host’s immune response, duration of infection, virulence, and infectious dose of the parasite. The main symptoms that may appear during infection are nausea, diarrhea (followed by dehydration), abdominal pain, vomiting, and flatulence [3,4]. During the life cycle, the protozoan forms a mobile trophozoite and an invasive cyst [5]. The trophozoite is the vegetative form and replicates in the host’s small intestine. The eight flagella provide motility, and the ventral disk mediates attachment to the intestinal wall, where it gains its nutrients. More distally, in the small intestine, and even extending to the large intestine, the trophozoite encysts into a cyst that is environmentally stable and can be transmitted to the next host through the fecal–oral route [6]. Flagellar motility is a key factor in *Giardia* pathogenesis and colonization of the host small intestine [7]. It is a crucial process for initiating and maintaining infection in the gut, permitting the parasite to avoid peristaltic flow and consequently search for suitable sites to attach to the intestinal lumen [8]. The presence of preservatives in food may therefore affect the parasite’s survival in the human digestive system. The weakening of the motility of trophozoites may lead to their removal from the human digestive system.

*Cryptosporidium* spp., *G. intestinalis*, and *Toxoplasma gondii* are the most common food-borne protozoa. The main factors contributing to the occurrence of foodborne diseases are raw food, excessive temperature, improper storage, improper handling, undercooking, and cross-contamination. The greatest threats come from food of animal origin, fresh produce, and ready-to-eat products, which are new food safety concerns [9].

The sensitivity of pathogens to physical and chemical factors is the basis for developing measures to reduce the incidence of the population. Sensitivity to physical factors plays an essential role in the disinfection of products that either should not be treated with chemicals (fresh fruit, milk, etc.) or are exposed to low concentrations of chemicals that do not affect all types of pathogens (e.g., chlorination water, salting or marinating meat, fish, and vegetables) or have a very high resistance to chemicals, and disinfection is mainly done by physical methods (e.g., helminths). Sensitivity to drying can be both main (spices, tea, dried fruit, dried meat, and fish) and additional (dry fruit concentrates for the production of juices and juice drinks, powdered milk, powdered eggs, etc.) measures to reduce risk when processing products of plant and animal origin. Temperature sensitivity is an essential criterion for preventing the spread of biological agents among humans. High temperatures are the basis of disinfection in technological processes of product processing. Low temperatures play an essential role in the disinfection and storage of products. However, some pathogens successfully survive and reproduce at temperatures close to zero [10].

Several methods are available to detect the presence of *G. intestinalis*. Culturing intestinal protozoa allows learning about, among others, the biology of parasites, their growth rate, virulence factors, susceptibility to new drugs, and the development of resistant strains [11]. Although cultivating human intestinal protozoa is useful for detection and diagnostic purposes, routine culture techniques were not established for *Giardia* spp. in the clinical diagnostic laboratory. Cultivation of *Giardia* spp. is applied in the research laboratory for many types of studies requiring many trophozoites. The *Giardia* spp. is grown in the monoxenic and axenic type of culture system. In the monoxenic system, the parasite has been grown in the presence of a single additional flora organism species. In axenic culture, the parasite has been grown without any other accompanied alive cell. The most common and suitable used medium for *Giardia* axenic culture is Diamond’s medium “TYI-S-33” [12].

The developed molecular methods demonstrate considerable specificity and sensitivity compared with microscopy and antigen detection methods. Compared with conventional methods, methods based on detecting intestinal protozoa DNA are faster, have higher sensitivity and specificity, can detect multiple parasites simultaneously, and can quantify and genotype parasitic DNA [13]. Another added advantage of these tools is the direct detection and molecular characterization of the identified parasite [14]. Some studies suggest PCR detection of these protozoa is more sensitive than methodologies such as microscopy and ELISAs, while others have noted a significant lack of sensitivity. This variation in reported sensitives may be linked to factors such as DNA extraction methods, the presence of PCR inhibitors, gene region, and the variety of sample types being analyzed, e.g., fecal, soil, and water. A standardized molecular detection method that is rapid, affordable, sensitive, and specific across a range of sample types still needs to be discovered [15].

The small number of cases of giardiasis detected in Poland may be due to the small number of samples tested or the influence of specific factors/methods used to increase food and water safety. Food safety is one of the most significant challenges in today’s world. It is essential to maintain appropriate standards when processing food.

The study aimed to determine the influence of selected factors on the survival assessment and detection of *G. intestinalis* DNA in axenic culture. Observations from this study will allow assessing whether the factors used act as potential inhibitors of the PCR reaction.

## 2. Materials and Methods

The research material was an axenic culture of the *G. intestinalis* strain obtained from the Institute of Maritime and Tropical Medicine in Gdynia. The TYI-S-33 medium was prepared for culturing. To 250 mL of deionized water was added 250 mg of KH_2_PO_4_, 150 mg of KH_2_PO_4_, 500 mg of L-cysteine, 500 mg of NaCl, 100 mg of l-ascorbic acid, 5.7 mg of ferric ammonium citrate, 187.5 mg of bovine bile, 7.5 g Bacto Peptone, and 5 g glucose (Sigma-Aldrich, St. Louis, MO, USA). All ingredients were mixed thoroughly, and the pH of the solution was adjusted to 7.0. The medium was then sterilized using syringe filters with a pore size of 0.2 µm. After filtration, 25 mL of calf serum, 0.25 mL of gentamicin solution (Krka d.d., Novo Mesto, Slovenia), and 0.9% NaCl (2 mL of gentamicin 40 mg/mL + 7 mL of 0.9% NaCl) were added. The medium was then poured into 20 mL tubes. The culture was passaged every 7 days. Before passaging *G. intestinalis*, the medium was heated to 37 °C.

The survival of *G. intestinalis* and the detection of protozoan DNA were assessed after exposure to various factors (Table 1).

Available physical and chemical agents were used, which can be used to increase the biological safety of, e.g., the environment and food as disinfectants or preservatives. In the case of some substances, solutions were prepared with the most commonly used concentrations, e.g., in medicine, food, or cosmetics. The analysis of the influence of each factor was repeated 4 times, and the study control was pure *G. intestinalis* culture. The criterion of a positive result was the observation of trophozoite movement in at least one preparation in the microscopic method, while in the real-time PCR method it was the detection of DNA in at least one reaction.

The microscopic method was used to assess the survival of *G. intestinalis* after being subjected to selected factors. Laboratory diagnostics for detecting protozoan DNA was performed by real-time PCR using an open system—Light Cycler 480 II (Roche, Basel, Switzerland). The following reagents were used in the open system: manual isolation kit (Roche, High Pure PCR Template Preparation Kit—catalog no. 11796828001); DNA Process Control Kit also containing reagents to prepare Master Mix reaction mix (Roche, DNA Process Control Kit Trial Pack—Cat. No. 07339666001); and primers and controls (TIBMOLBIOL, Berlin, Germany, LightMix Modular *Giardia*—catalog no. 07979754001).

In the open system, the nucleic acid is isolated in the first stage of the study using the Roche manual isolation kit. The process begins with the preparation of the analyzed material. The *Giardia* culture (200 µL) is centrifuged at 7000 rpm, and then the cell pellet is resuspended in PBS (200 µL). Then, an internal control (20 µL), Binding Buffer (200 µL), and Proteinase K (40 µL) are added. After 10 min of incubation at 70 °C, isopropanol (100 µL, WARCHEM, Warsaw, Poland) is added. The sample volume is then transferred to the column (High Filter Tube) and centrifuged for 1 min at 11,000 rpm. In the next step, an inhibitor (Inhibitor Removal Buffer; 500 µL) is added to remove PCR inhibitory impurities. The sample is centrifuged again. The washing process by adding a buffer (Wash Buffer; 500 µL) aims to purify RNA from various salts, proteins, or other contaminants. The sample is then centrifuged again, and the rinsing step is repeated. After centrifugation, the sample is dried by short spinning at the maximum speed of 13,000 rpm for 10 s. In the last step, the bound nucleic acid is eluted by adding an appropriate buffer heated to 70 °C (Elution Buffer; 200 µL) and centrifuging for 1 min at 11,000 rpm. The obtained isolate (eluate) contains the tested genetic material for further diagnostics. The following research stage is a one-stage real-time PCR reaction in the LightCycler 480 II apparatus. DNA is replicated with the participation of polymerase according to the following scheme:

- Initial denaturation—separation of double-stranded DNA under the influence of high temperature;
- Attachment of primers/probes (annealing);
- Amplification (elongation).

The reaction follows the profile programmed on the device. The real-time PCR method enables the detection of amplified fragments in real time.

The LightCycler 480 II instrument measures the emitted fluorescence and plots the result. All obtained results from the reading channels are analyzed and compared with positive and negative controls.

## 3. Results

In the conducted studies, which analyzed the influence of 22 selected factors on the survival of G. intestinalis, no effect was observed in the microscopic method for 5 substances (22.7%): maltodextrin, sodium citrate E331, calcium lactate E327, propylene glycol, and sodium bicarbonate. On the other hand, the lack of trophozoite movement was noted in the case of 17 different factors (77.3%). On the other hand, when assessing the influence of all factors on the detection of G. intestinalis DNA using the real-time PCR method, 19 factors (86.4%) showed no effect on the course of the reaction. Only in the case of three substances (13.6%) was their influence on detecting the presence of protozoan genetic material found. The addition of sodium hypochlorite, formalin, and sodium bicarbonate resulted in the absence of Giardia DNA. All CP values obtained in the real-time PCR method were in the range of 21.48–28.05. The obtained results are presented in Table 2.

In the case of maltodextrin, sodium citrate E331, calcium lactate E327, and propylene glycol, the same results were obtained in both methods as for pure *G. intestinalis* culture, i.e., the movement of trophozoites was observed, and the genetic material of the protozoan was detected. Due to the influence of UV radiation, various temperatures, ethanol, disinfectants, citric acid E330, sodium benzoate, and 10% NaOH, the microscopic method did not show the movement of trophozoites. At the same time, a positive result was obtained in real-time PCR. In turn, in the case of sodium bicarbonate, the movement of trophozoites was observed, but the presence of *Giardia* DNA was not found.

## 4. Discussion

The analysis shows that only four of the applied factors did not affect the survival and detection results of *G. intestinalis* DNA.

Considering UV radiation, no trophozoite movement was found in the microscopic method, while the real-time PCR showed the protozoan genetic material’s presence. Li et al. [16] observed that some *G. lamblia* trophozoites could survive or be reactivated after exposure to UV radiation up to 10 mJ/cm^2^. Evidence of survival or reactivation at the 20 and 40 mJ/cm^2^ UV fluencies was inconclusive, while at 100 mJ/cm^2^, there was no evidence of survival or reactivation. This may impact the criteria used by the drinking water and wastewater industry to ensure the safe reduction in *G. lamblia* cysts in UV disinfection processes [16]. In turn, other studies on UV disinfection of G. lamblia cysts in water concluded that UV disinfection at practical doses achieves significant (much greater than 4 logs) inactivation of *G. lamblia* cysts in water without evidence of DNA repair leading to reactivation of infectivity [17]. Adeyemo et al. [18], in their research concerning the efficiency of chlorine and UV in the inactivation of *Cryptosporidium* and *Giardia* in wastewater, observed that *Giardia* is less resistant to UV irradiations than *Cryptosporidium*. UV irradiation has more effect on *Giardia* than chlorine. This is due to the killing effect exhibited by UV on Giardia because of its impact on its DNA. UV radiation of 50 mJ/cm^2^ at 280 nm can destroy only *Giardia cysts* from the feces of infected patients to a maximum of 75% [19]. Einarsson et al. [20] examined the response to ultraviolet (UV) radiation, a natural stressor of the intestinal protozoan parasite *G. intestinalis*, and observed that UV radiation of 10 mJ/cm^2^ was effective in killing *Giardia* cysts. The UV action on trophozoites induces DNA replication to stop. Active DNA replication and DNA repair may explain why UV light does not kill trophozoites and encapsulates cells as effectively as nonreplicating cysts. UV radiation induces minor overall changes in gene expression in *Giardia*, but cysts show a stronger response than trophozoites [20].

Analyzing the influence of temperature, the lack of trophozoite movement in the microscopic method and a positive reaction to the presence of *Giardia* DNA in the real-time PCR method were also shown. Utaaker et al. [21], by examining the survival of *Giardia* cysts on lettuce, observed that *Giardia* cysts survive well when kept moist and refrigerated. Survival of *Giardia* cysts was abrogated on lettuce at room temperature. Indeed, an almost 50% die-off of *Giardia* cysts was recorded within the first 24 h. *Giardia* cysts were less robust than *Cryptosporidium* oocysts and would be unlikely to survive under ambient storage conditions on-farm, during the sale, or at home. However, if refrigerated, some contaminating *Giardia* cysts may remain viable and threaten the consumer [21].

Wickramanayake et al. [22], comparing the effect of ozone and storage temperature on cyst survival, *G. lamblia*, and *Giardia muris*, found that the cyst viability was similar over the 25-day storage period ranging from −6 to 37 °C, and the optimal temperature for their long-term survival is around 5 °C. Ozone was highly effective in inactivating the cysts of both *Giardia* species, with *G. muris* being consistently more resistant than *G. lamblia* at pH 7 and 5 °C and 25 °C [22]. In an experiment involving the influence of several factors on *Giardia* excystation in vitro, it was shown that temperature, pH, time, and medium impact the level of excystation achieved. Excystation was adopted as the criterion of viability. The effect of storage at −13, 8, 21, and 37 °C and exposure to boiling water on the survival of *Giardia* cysts was investigated. Storage at 8 °C allowed for the most extended survival of the cysts, 77 days. Cysts stored at 21 °C were viable for 5 to 24 days, while those at 37 °C never survived for more than 4 days. Freezing and thawing of cysts resulted in almost complete loss of viability, although the low level of viability (<1%) was maintained for at least 14 days. Cysts exposed to boiling water were immediately unable to excistate [23]. In turn, El Mansoury et al. [24] assessed the influence of different degrees of temperature and salinity on the viability and infectivity of *G. lamblia* and *C. parvum* at different storage times. It was shown that boiling the protozoa for one minute minimized their viability to less than 1% and rendered them noninfectious, while exposure to 4 and −4 °C for up to seven days kept them viable and infectious. However, salinity was found effective at high concentrations (50 ppt) or extended storage times at lower concentrations [24].

In the presented study, the use of sodium hypochlorite influenced the results of both methods, making it impossible to observe the trophozoite movement and detect *Giardia* DNA. Jarroll et al. [25] assessed the effect of chlorine concentration under various conditions on the viability of *G. lamblia* cysts. The excretion capacity of *Giardia* cysts was adopted as a criterion for viability. The influence of temperature (25, 15, and 5 °C), pH (6, 7, and 8), contact time of chlorine with the cyst (10, 30, and 60 min), and chlorine concentration (1 to 8 mg/L) were investigated. Cyst survival increased with increasing buffer pH. In combination with chlorination, water temperature proved essential for cyst survival. At 25 °C, 1.5 mg/L exposure for 10 min killed all cysts at pH 6, 7, and 8. At 15 °C, 2.5 mg of chlorine per liter for 10 min killed all cysts at pH 6, but at pH 7 and 8, a small number of cysts remained viable after 30 min, but not after 60 min. At 5 °C, 1 mg of chlorine per liter for 60 min did not kill all cysts at any pH tested. At this temperature, 2 mg of chlorine per liter killed all cysts after 60 min at pH 6 and 7 but not at pH 8. A 4 mg/L chlorine concentration killed all cysts at all three pH values after 60 min but not 30 min. The 8 mg/L chlorine concentration killed all *Giardia* cysts at pH 6 and 7 after contact for 10 min and at pH 8 after 30 min [25]. Kim et al. [26] observed that the killing rate was initially fast when sodium hypochlorite containing 5 or 10 ppm of chlorine was used, but disinfection slowed down. A 3-log reduction could not be achieved even after 2 h. The disinfection efficiency was also reduced at lower temperatures [26].

This study noted the influence of formalin on the test results. Both methods obtained negative results. On the other hand, 70% ethanol did not affect the detection of protozoan DNA. Kuk et al. [27], when examining the effect of stool storage conditions to preserve *Giardia* DNA, found that all stool samples containing cysts that were stored at room temperature, +4 and −20 °C, and in 70% alcohol and 2.5% potassium dichromate, obtained a band of 384 bp specific for *G. intestinalis*. However, this strip was not produced by the samples stored in 10% formaldehyde. In the case of fecal samples containing trophozoites, the same *G. intestinalis*-specific band was obtained only from samples stored in 2.5% potassium dichromate for up to one month [27]. Bezagio et al. [28] also noted that formaldehyde might have a negative impact on the detection of *Giardia duodenalis* by molecular methods. DNA samples were tested for glutamate dehydrogenase (GDH) and β-giardine (βg) genes. GDH and βg genes were not detected when the sample was treated with formaldehyde [28].

Marchin et al. [29] suggest that the disinfectant’s effect depends on the *Giardia* wall’s thickness, and therefore lasts longer at a lower temperature.

Analyzing the influence of sodium bicarbonate on trophozoite movement and detection of *Giardia* DNA, it was found that it influences the course of the PCR reaction, resulting in the lack of detection of genetic material. Feely et al. [30] suggest that excystation can be stimulated by a bicarbonate–phosphate medium. In this study, *Giardia muris* cysts were incubated briefly in an aqueous-inducing medium of 0.1 M potassium phosphate with 0.1, 0.2, or 0.3 M sodium bicarbonate. High rates of excystation (91.1–96.7%) were recorded within 5 min after placing the cysts in the TYI-S medium [30]. Sodium bicarbonate can be used to prepare the medium for in vitro excystation of *G. lamblia* cysts [31].

Wilke et al. [32] suggest that the lack of PCR amplification from a proportion of the fecal samples can be associated with the storage medium used for the samples. They applied four different, commonly used storage media to investigate their effects over time on subsequent PCR amplification of DNA from *Giardia* cysts in stool samples. Microscopic examination of the samples and real-time PCR were used to analyze 7 samples over 3 months. Storage in ethanol or potassium dichromate at 4 °C gave the best results. If the immunomagnetic separation was used before PCR (as may be appropriate for samples with low cyst numbers), then storage in potassium dichromate gave the best results [32]. A *G. intestinalis*-specific 384 bp band was obtained from all of the cyst-containing stool samples stored at RT, +4 °C and −20 °C and in 70% alcohol and 2.5% potassium dichromate; however, this band was not produced by samples that were stored in 10% formaldehyde. Moreover, for the stool samples containing trophozoites, the same *G. intestinalis*-specific band was only obtained from the samples that were stored in 2.5% potassium dichromate for up to one month. The most suitable storage condition for stool samples to permit the isolation of *G. intestinalis* DNA is in 2.5% potassium dichromate; under these conditions, stool samples may be stored for one month [27].

The study detected no DNA when formalin was added to the culture—storage of samples in formalin results in nucleic acid cross-linking and DNA fragmentation [33]. Formalin damages DNA in three ways: fragmentation, base modification, and cross-linkage within the DNA itself or between DNA and proteins [34]. Similarly, there was no positive PCR reaction when sodium hypochlorite was used. Fischer et al. [35] observed that DNA exposure to sodium hypochlorite prevents PCR amplification of a 76 bp amplicon. Decontamination is defined as the degradation, denaturation, or inactivation of the amplification of nucleic acids [35].

There are no studies on the influence of some of the selected physical and chemical factors on the survival assessment and detection of *G. intestinalis* DNA. It is necessary to continue research on the impact of various factors on detecting protozoa due to the small number of factors used in the presented study.

This study has some limitations. In routine diagnostics, trophozoites and parasite cysts may be detected in the feces of people infected with *Giardia lamblia*. In formed feces, cysts predominate. However, in diarrheal and watery feces, trophozoites are the dominant form. Our research focuses on *Giardia* trophozoites only. In future studies, the impact of the studied factors on the survival assessment and detection of *Giardia intestinalis* DNA in axenically cultured cysts should be determined. The examination of cysts and trophozoites would give a complete picture of the influence of the investigated factors. The involvement of an in vitro encystation protocol should be optimized for the strain used in our study.

## 5. Conclusions

Most of the factors are used to affect the survival rate of protozoa. It seems that standard disinfection methods using UV, ethanol, or various disinfectants are sufficient to reduce survival. Boiling water or using preservatives such as citric acid can also contribute to this. In turn, detecting DNA in most samples may suggest the need, for example, to extend the period of influence of a specific factor. Therefore, the conducted research should be extended.

The research results indicate a much higher sensitivity of genetic methods used in laboratories than microscopic techniques. Modern diagnostic methods significantly increase the possibility of detecting parasites by detecting their genetic material. In the case of infection, determining the presence of *Giardia intestinalis* based solely on the microscopic method may give false-negative results. Trophozoites die very quickly in clinical material. Using more sensitive techniques, such as PCR, increases the possibility of detecting parasites. The development of a protozoan detection method is needed for food safety monitoring.

Prevention of *G. intestinalis* infections, among others, by ensuring food and water safety is a crucial issue affecting public health. Using preservatives or various disinfection methods affects the survival of protozoa and thus reduces their spread. The main route of infection with *Giardia* is the alimentary canal, so the risk of giardiasis is reduced by increasing food safety. This may contribute to establishing measures to reduce outbreaks of parasitic diseases associated with contaminated food.

## Figures and Tables

**Table 1:** Factors used to evaluate their effect on survival and DNA detection of *Giardia intestinalis*.

Factor	Solution	Time/Volume
UV 15 min	-	15 min
UV 30 min	-	30 min
temp. 100 °C	-	10 min
temp. 2–8 °C	-	24 h
temp. −20 °C	-	24 h
ethanol 70% p.a. (POCH, Gliwice, Poland)	-	100 µL
ethanol 96% p.a. (POCH, Gliwice, Poland)	-	100 µL
disinfectant 1 (Line Antybakteria 70)—for hands, surfaces, and objects with antibacterial and antifungal properties (SYL-CHEM, Bydgoszcz, Poland)	-	100 µL
disinfectant 2 (Dezynmax TZF)—for hands, surfaces, and devices with virucidal, bactericidal, and fungicidal activity (TZF POLFA, Warsaw, Poland)	-	100 µL
disinfectant 3 (Trisept TZF)—for hands with virucidal activity (TZF POLFA, Warsaw, Poland)	-	100 µL
benzyl alcohol 100% (Biomus, Lublin, Poland)	2%	100 µL
sodium hypochlorite 15% (Biomus, Lublin, Poland)	-	100 µL
formalin 35–38% (Hardron Scientific, Kielce, Poland)	10%	100 µL
citric acid E330 (AGNEX, Białystok, Poland)	30%	100 µL
maltodextrin (AGNEX, Bialystok, Poland)	10%	100 µL
sodium citrate E331 (AGNEX, Bialystok, Poland)	5%	100 µL
calcium lactate E327 (AGNEX, Bialystok, Poland)	1%	100 µL
propylene glycol (Hardron Scientific, Kielce, Poland)	10%	100 µL
sodium benzoate (Hardron Scientific, Kielce, Poland)	65%	100 µL
sodium bicarbonate (Hardron Scientific, Kielce, Poland)	5%	100 µL
10% NaOH (Sigma-Aldrich, St. Louis, MO, USA)	-	100 µL

**Table 2:** Influence of selected factors on survival and detection of *Giardia intestinalis* DNA.

Factor	The Result of the Microscopic Method	The Result of the Real-Time PCR Method
pure *Giardia intestinalis* culture	+	+
UV 15 min	-	+
UV 30 min	-	+
temp. 100 °C	-	+
temp. 2–8 °C	-	+
temp. −20 °C	-	+
ethanol 70% p.a.	-	+
ethanol 96% p.a.	-	+
disinfectant 1	-	+
disinfectant 2	-	+
disinfectant 3	-	+

+ trophozoite movement/DNA presence; - no trophozoite movement/no DNA present.

## Data Availability

The data presented in this study are available on request from the corresponding author. The data are not publicly available due to privacy or ethical restrictions.
